# Assessing the Impact of Different Mixing Strategies on Genomic Prediction Accuracy for Beef Cattle Breeding Values in Multi-Breed Genomic Prediction

**DOI:** 10.3390/ani15162463

**Published:** 2025-08-21

**Authors:** Le Zhou, Lin Zhu, Fengying Ma, Mingjuan Gu, Risu Na, Wenguang Zhang

**Affiliations:** 1 College of Animal Science and Technology, Inner Mongolia Agricultural University, Hohhot 010018, China; zxcvbnm8880314@163.com (L.Z.);; 2 Key Laboratory of Animal Genetics, Breeding and Reproduction of the Inner Mongolia Autonomous Region, College of Animal Science and Technology, Inner Mongolia Agricultural University, Hohhot 010018, China

**Keywords:** genomic estimated breeding value, single-step genomic best linear unbiased prediction, weighted genomic best linear unbiased prediction, multi-breed

## Abstract

Although genomic selection can accelerate livestock breeding, its application in many countries is hindered due to the limited size of reference populations. To address this issue, researchers have explored methods of combining multiple breeds to create reference populations, aiming to enhance the accuracy of genomic prediction. The main objective of this study was to evaluate the impact of the construction of mixed reference populations at different genetic distance levels on the accuracy of multi-breed genome prediction in multi-breed beef cattle populations using three evaluation methods: GBLUP, ssGBLUP, and wGBLUP. In order to study the effect of genetic correlation on multiple populations and to resolve the optimal mixing ratio, we considered six scenarios, including (1) population A as the main body, where the nearest 10% of individuals in populations B and C were added; (2) population A was the main body, where the 15% of individuals with the closest genetic distance in groups B and C were added; and (3) population A as the main body, where the 20% of individuals in populations B and C with the closest genetic distance were added. Our results suggest that the wGBLUP model can be enhanced when the mixing ratio is 15%, and the wGBLUP model shows higher accuracy in predicting populations with different LD decay patterns. Among them, whether combined with PopB or PopC, the wGBLUP model shows better prediction ability than the GBLUP and ssGBLUP models. However, when the mixing ratio is 10% or 20%, the accuracy of the three models is less than 15%, and the wGBLUP and ssGBLUP models show high and stable accuracy. Our results highlight the importance of considering the proportion of mixing between different populations when using genetic assessment models to predict accuracy, especially for endemic beef cattle breeds with different genetic structures and LD patterns and limited resources. However, this study also has some limitations. First, the determination of the optimal mixing ratio still needs further exploration, especially for populations with different genetic structures and LD patterns. Second, future studies can introduce more advanced models to further improve prediction accuracy.

## Simple Summary

Genomic selection (GS) accelerates livestock breeding but is limited by small reference populations in many countries. This study evaluated the impact of constructing mixed reference populations at different genetic distances on multi-breed genome prediction accuracy in beef cattle using GBLUP, ssGBLUP, and wGBLUP methods. Six scenarios with varying mixing ratios were considered. The results showed that the wGBLUP model had higher accuracy when the mixing ratio was 15%, especially for populations with different LD decay patterns. However, at 10% and 20% mixing ratios, wGBLUP and ssGBLUP had lower but stable accuracy. This study highlights the importance of optimizing mixing ratios for accurate genetic prediction in multi-breed beef cattle populations with limited resources.

## 1. Introduction

Genomic selection (GS) has emerged as a pivotal breeding method, widely adopted in both animal and plant breeding domains, thanks to the advent of single-nucleotide polymorphism (SNP) chips and re-sequencing technologies. Compared to traditional breeding methods, GS has achieved significant improvements in selection accuracy and the rate of genetic progress, particularly for traits that are difficult to enhance through conventional approaches, such as feed efficiency, reproductive traits, health traits, and meat quality [1]. The core mechanism of GS lies in utilizing SNPs across the entire genome to explain genetic variance and accurately estimate SNP effects, a process that heavily relies on the linkage disequilibrium (LD) between SNPs and quantitative trait loci (QTL). In this way, GS has successfully overcome the limitations of traditional breeding methods, which were characterized by low accuracy and slow genetic progress. This is especially evident in species such as pigs and dairy cows, where GS has significantly improved breeding outcomes by increasing selection accuracy and accelerating genetic progress [2,3]. However, the prediction accuracy of GS is largely influenced by the size of the reference population and the strength of LD between SNPs and QTL. To achieve accurate genomic prediction, a large reference population with both phenotypic and genotypic information is typically required. Yet, building such a large training set remains challenging for some breeds or traits. For example, in small population breeds like the Dutch Red dairy cattle, the application of genomic prediction is hindered by the lack of a sufficiently large reference population, resulting in a significant delay in the release of genomic estimated breeding values (GEBVs) compared to breeds with large reference populations [relatedness between numerically small Dutch Red dairy cattle populations and possibilities for multi-breed genomic prediction]. To address this issue, researchers have attempted several remedial measures, such as using SNP effects from other breeds for across-breed prediction or integrating data from multiple breeds for multi-breed prediction to improve the accuracy of SNP effect estimation [4]. However, these strategies have not been very effective. The accuracy of across-breed prediction is usually low, and combining data from multiple breeds does not significantly enhance prediction accuracy. This is due to the population-specific nature of SNP-QTL associations and the inconsistency of LD between different breeds, which severely limits the application of these strategies in small or distantly related breeds. Moreover, attempts to pool animals from related breeds have also failed, due to the lack of persistent associations between SNPs and QTLs or the inconsistency of LD between SNPs and QTLs in distantly related genetic groups [5,6]. This limitation is particularly evident in the beef cattle industry. Major breeds, such as Simmental, have widely implemented GS, while minor breeds (such as Red Angus/Japanese Black) face challenges of delayed GEBV release and incomplete trait coverage due to insufficient reference populations. Estimating the breeding values of the next generation’s optimal parental animals is a primary goal of animal breeding programs [7]. Therefore, further exploration of strategies to improve the accuracy of multi-breed prediction is needed to promote the universal application of GS in genetically diverse populations.

Multi-breed/across-breed genomic evaluation has shown great application potential in livestock breeding, especially in hybrid breeding and commercial production. By integrating reference populations from multiple breeds, this technology can theoretically enhance the accuracy of genomic prediction, particularly for breeds with small reference populations. Studies have shown that sharing 300,000 SNP markers can achieve relatively good across-breed prediction results among closely related breeds (such as Bos taurus cattle breeds). However, for distantly related breeds (such as Bos indicus), prediction accuracy is often difficult to improve due to differences in linkage disequilibrium (LD) patterns and population-specific causal mutations. The main advantage of multi-breed genomic evaluation is its ability to expand the selection pool and increase selection intensity. It is particularly beneficial for predicting breeding values of crossbred offspring and composite breeds. It can support across-breed selection, provide more data resources for small population breeds, optimize hybrid breeding strategies, and improve the prediction accuracy of breeding values in F1 and F2 generations [8,9]. However, this method still faces significant challenges. First, genetic differences between breeds are the main limiting factor. For distantly related breeds (such as Bos indicus), prediction accuracy is difficult to improve due to the population-specific nature of SNP-QTL associations and differences in LD patterns [10]. Second, the composition of the reference population needs to be carefully designed. Over-representation of data from dominant breeds can actually reduce prediction accuracy for minor breeds. For example, in a study on Australian Red cattle, prediction accuracy was compromised when the proportion of Holstein cattle in the reference population was too high [11]. Although the application of whole-genome sequencing data has brought new opportunities for multi-breed evaluation, only a slight increase in prediction accuracy has been observed in cattle and sheep so far. This suggests that technical bottlenecks such as genetic background differences and data integration complexity still exist.

In multi-breed/across-breed genomic evaluation, genetic correlation and linkage disequilibrium (LD) patterns together form the core determinants of prediction accuracy. The underlying mechanism is manifested in three key aspects: First, the heterogeneity of allelic substitution effects across breeds leads to lineage-specific QTL effects. Second, the degree of breed differentiation (such as the independent evolution of Bos indicus and Bos taurus for 600,000 to 800,000 years) directly determines the conservation of the LD phase. Third, selection pressures and genetic drift together shape the population-specific nature of SNP-QTL linkage relationships. To achieve high prediction accuracy in multi-breed populations, it is necessary to have high linkage disequilibrium (LD) between markers and QTLs within each breed, as well as high consistency in the genotype phase between markers and QTLs across different breeds [12,13]. This consistency depends on allele frequency differences and relatedness. If the LD phase consistency between breeds is high, the likelihood of the same LD phase between markers and QTLs across breeds is greater, which may enhance the accuracy of across-breed prediction. This theoretical framework explains why high-density chips (such as the 777 K BovineHD) can improve prediction performance compared to traditional 50 K chips—by increasing marker density to capture more stable LD blocks. Whole-genome sequencing (WGS) technology goes even further, breaking through the limitations of SNP-QTL linkage dependency by directly locating causal mutations [14,15]. However, selection and genetic drift can lead to different LDs between breeds or opposite allelic phases of SNPs and QTLs, further complicating predictions. This is especially true for cattle with both Bos indicus and Bos taurus ancestry. These two subspecies diverged around 600,000 to 800,000 years ago, and their multi-subspecies populations are common in tropical beef production systems, making across-breed prediction even more challenging [16]. Despite the new opportunities brought by the development of high-density genotyping arrays (such as the BovineHD chip) and WGS for multi-breed evaluation, the advantages of these technologies in multi-breed prediction still need to be further explored. Traditional SNP chips, with their insufficient marker density, struggle to capture persistent LD patterns across breeds. WGS data, by directly detecting causal mutations, holds the potential to reduce reliance on SNP-QTL linkage relationships. However, it is still limited by the insurmountable deep genetic divergence between distantly related breeds. This essentially reflects the evolutionary biological constraints that genomic selection techniques face when applied across species scales.

Significant progress has been made in multi-breed/across-breed genomic evaluation, with models such as GBLUP, ssGBLUP, and wGBLUP each demonstrating unique advantages. GBLUP serves as the foundational model, integrating whole-genome SNP information through the genomic relationship matrix (GRM) to provide essential support for the estimation of genomic breeding values. However, its assumption of equal effects for all variants may limit the ability to identify important functional variants in across-breed prediction [17]. In contrast, ssGBLUP integrates pedigree, genomic, and phenotypic information, making it particularly suitable for small populations. It can more effectively handle large-effect QTLs and polygenic effects, thereby significantly improving prediction accuracy. wGBLUP further enhances the model’s ability to identify important functional variants by weighting the genomic relationship matrix, assigning greater weight to preselected sequence variants [18]. Recent studies have shown that defining relationships between breeds using haplotypes rather than individual SNPs, or integrating intermediate omics data, can effectively break through the bottleneck in across-breed prediction and further improve prediction accuracy [19,20]. Moreover, the metafounder model, as a multi-breed extension of ssGBLUP, provides new ideas for across-breed evaluation by establishing genetic connections between breeds. However, the prediction accuracy of this model in uncharacterized breeds still needs to be improved [21]. In practice, the application of these models in complex hybrid systems, such as tropical cattle (Bos indicus × Bos taurus), still faces challenges. Due to limited breed composition information and incomplete pedigree data, the direct application of ssBLUP and the metafounder model is difficult [22]. This highlights the urgent need to develop new methods that can effectively handle incomplete pedigrees and unknown breed compositions. Overall, GBLUP, ssGBLUP, and wGBLUP have significantly improved the prediction accuracy of multi-breed evaluation by integrating various sources of information, providing strong support for animal breeding. However, further research and comparison are still needed for the application of these models in small populations and complex breed backgrounds to fully realize their potential in across-breed evaluation.

Therefore, this study employed three evaluation methods—GBLUP, ssGBLUP, and wGBLUP—to investigate the comparative accuracy of multi-breed/across-breed genomic prediction in multi-breed beef cattle populations when constructing mixed reference populations at different genetic distance levels. To identify the optimal mixing ratio, six scenarios were considered, including (1) using population A as the main body and adding the nearest 10% of individuals from populations B and C; (2) using population A as the main body and adding the nearest 15% of individuals from populations B and C; and (3) using population A as the main body and adding the nearest 20% of individuals from populations B and C.

## 2. Materials and Methods

### 2.1. Data Simulation

First, the pedigree and genomic data were simulated using QMSim Version 1.10 [23]. To investigate the impact of different linkage disequilibrium (LD) patterns on the accuracy of genomic estimated breeding values (GEBVs) in beef cattle populations, the initial historical population (HP) was simulated into three distinct beef cattle populations (PopA, PopB, and PopC) based on three different LD patterns. Second, the final breeding animals from these populations were used as candidates for alternative selection schemes. QMSim was used to simulate five replicates, i.e., five sets of final breeding animals. Within each replicate, the breeding schemes studied selected the first batch of breeding animals from the same population, although different schemes could use different groups of breeding animals. To save data storage space without compromising computational speed, only the genotypic and phenotypic data of animals in the 9th and 10th generations were simulated. The phenotypes were sampled from a normal distribution with a mean of 0 and a variance of 1, with the overall mean being the only fixed effect. Table 1 summarizes the population structure and parameters of the simulation process.

#### 2.1.1. Population Structure

**Figure 1:**
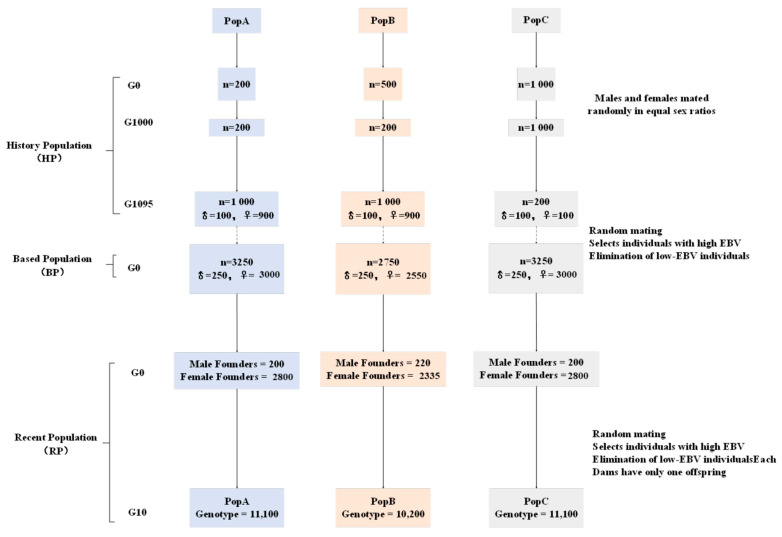
Visual presentation of the simulated data. PopA, PopB, and PopC experienced different phases of bottlenecks and expansions to generate differences in linkage disequilibrium. Founder animals were selected for the three populations and randomly mated for 10 generations, resulting in different population sizes, and the number of genotyped animals was selected.

In QMSim, the historical population (HP) was generated based on equal sex ratios, non-overlapping generations, random mating, no selection, and no migration. These conditions were used to simulate initial values of linkage disequilibrium (LD), mutations, and genetic drift, thereby mimicking the structure and extent of LD present in beef cattle populations [24]. The population structures of the three distinct beef cattle populations (including historical, base, and current population structures) were simulated in detail, as described in Figure 1.

(1) PopA simulated a linkage disequilibrium (LD) pattern that began with a stable population and then experienced continuous expansion. First, the historical population (HP) of PopA was simulated, consisting of an initial 200 individuals that remained at a constant size of 200 individuals over 1000 generations. In the subsequent 95 generations, the population size gradually expanded to 1000 individuals (100 males and 900 females) to establish an initial LD and mutation-drift equilibrium. Next, 250 males and 3000 females were randomly selected from the last generation of the historical population to expand the population size. All males and females were randomly mated, with each female producing one offspring per generation for 10 generations. Finally, 200 males and 2800 females were randomly selected from the expanded base population. These selected individuals were simulated over the most recent 10 generations, with each female producing one offspring per generation. The selection design was based on phenotypic performance and BLUP (EBV) methods. The replacement rates were 60% for bulls and 30% for cows. The missing record rate for bulls and cows was 0.05.

(2) PopB simulated a linkage disequilibrium (LD) pattern that began with an initial stable population and then experienced contraction, followed by expansion. First, the historical population (HP) of PopB was simulated, consisting of an initial 500 individuals that decreased to a stable population size of 200 individuals over 1000 generations. In the subsequent 95 generations, the population size gradually expanded to 1000 individuals (100 males and 900 females) to establish an initial LD and mutation-drift equilibrium. Next, 250 males and 2550 females were randomly selected from the last generation of the historical population to expand the population size. All males and females were randomly mated, with each female producing one offspring per generation for 10 generations. Finally, 220 males and 2335 females were randomly selected from the expanded base population. These selected individuals were simulated over the most recent 10 generations, with each female producing one offspring per generation. The selection design was based on phenotypic performance and BLUP (EBV) methods. The replacement rates were 50% for bulls and 30% for cows. The missing record rate for bulls and cows was 0.05.

(3) PopC simulated a linkage disequilibrium (LD) pattern that began with an initial stable population and then experienced expansion, followed by contraction. First, the historical population (HP) of PopC was simulated, consisting of an initial 1000 individuals that remained at a stable population size of 1000 individuals over 1000 generations. In the subsequent 95 generations, the population size gradually decreased to 200 individuals (100 males and 100 females) to establish an initial LD and mutation-drift equilibrium. Next, 250 males and 3000 females were randomly selected from the last generation of the historical population to expand the population size. All males and females were randomly mated, with each female producing one offspring per generation for 10 generations. Finally, 200 males and 2800 females were randomly selected from the expanded base population. These selected individuals were simulated over the most recent 10 generations, with each female producing one offspring per generation. The selection design was based on phenotypic performance and BLUP (EBV) methods. The replacement rates were 50% for bulls and 20% for cows. The missing record rate for bulls and cows was 0.05.

#### 2.1.2. Genome

The bovine genome assembly version ARS-UCD1.2 (https://jul2023.archive.ensembl.org/Bos_taurus/Info/Index (accessed on 7 May 2025)) was used to simulate the 29 pairs of autosomes in beef cattle, with a total length of 2715.85 centimorgans. The aim was to create a more realistic scenario by considering the true distances between markers and quantitative trait loci (QTL) sites. SNP markers were uniformly distributed and randomly generated, with a density of 50 k markers, representing diallelic loci with segregation. The number of SNPs on each chromosome was proportional to its size. The effects of markers on traits were assumed to be neutral. The whole genome consisted of 725 quantitative trait loci (QTL). The number of recombinations per Morgan was sampled from a Poisson distribution with a mean of 1, and crossovers were randomly placed along the chromosomes. In the final generation of the historical population (HP), a TLR effect for a production trait was introduced. This trait had a heritability of 0.42, similar to the heritability of birth weight in beef cattle [25]. In QMSim, the genetic effects for this trait were sampled from a gamma distribution with a shape parameter of 0.4. Over the 1095 generations of the HP, the mutation rate for both QTL and SNPs was 2.5 × 10^−5^, with the SNP mutation pattern set to “recurrent,” meaning mutations only switched between alleles without generating new mutation types. The detailed simulation parameters are summarized in Table 1.

#### 2.1.3. Genetic Evaluations

The simulated heritability was 0.42, and the phenotypic variance for the single trait was set to 1.0. The true breeding value (TBV) for each animal was calculated as the sum of the additive effects of the QTL, as follows:

(1) $${TBV}_{k}=\sum_{j=1}^{qtl}\beta_{j}\cdot Q_{kj},$$

where $\beta_{j}$ is the additive effect of ${QTL}_{j}$, and $Q_{kj}$ is the QTL genotype at locus j, coded as 0, 1, or 2, as the number of copies of a specified QTL allele is carried by an individual (k). The phenotypes ($y_{i}$) were simulated by adding a residual term sampled as εi ∼ N(0,σe2), where σe2 is the residual variance.

The EBVs were estimated for all individuals in the current population from Generations 9 to 10 based on phenotypic values and pedigree data. The best linear unbiased prediction (BLUP) of breeding values was obtained by Henderson’s mixed linear model. The BLUP predictor had the smallest prediction error variance among all possible linear unbiased predictors. The numerator relationship matrix (A) was used in the following mixed model equations to derive BLUP of random additive effects (including polygenes and QTL):

(2) $$\left[Z^{\prime }Z+A^{-1}\frac{\sigma_{e}^{2}}{\sigma_{a}^{2}}\right]\left[\hat{a}\right]=\left[Z^{\prime }y\right]$$

where y is the vector of phenotypic records, Z is the incidence matrix relating the records to the random additive effects (a), $\sigma_{e}^{2}$ is the residual variance, and $\sigma_{a}^{2}$ is the additive genetic variance. The mixed model equations were solved using the conjugate gradient method.

### 2.2. Genotype Quality Control

The PLINK v1.9 software [26], a widely used toolset for whole-genome association and population-based linkage analyses, was employed to further filter SNPs based on the following criteria: minor allele frequency (MAF) < 0.05, genotype call rate < 0.10, individual call rate < 0.10, and Hardy–Weinberg equilibrium < 1 × 10^−5^. After genotype quality control, PopA, PopB, and PopC retained 52,676, 53,340, and 52,081 segregating SNPs with MAF > 0.05, respectively, for subsequent analyses. The simulation assumed that QTL allele effects were identical across all breeds, but the frequencies of QTL varied among breeds, resulting in differences in the variance explained by each breed.

### 2.3. Scenarios

This study examined various scenarios to evaluate the impact of three different levels of genetic distance (Top10%, Top15%, and Top20%) between populations and the mixing ratio on the accuracy of GEBVs (genomic estimated breeding values). Specifically, individuals ranking in the top 10%, 15%, and 20% from populations B and C were selected and combined with all individuals from population A. For example, if population B had 1000 animals, then the top 100 (10%), 150 (15%), or 200 (20%) animals from population B were added to population A. Similarly, individuals from population C in the corresponding proportions were also added to the combined reference population. In this way, six different mixed combinations were constructed.

(1) A+10%B: With population A as the main body, individuals with the top 10% genetic distance from population B were added;
(2) A+15%B: With population A as the main body, individuals with the top 15% genetic distance from population B were added;
(3) A+20%B: With population A as the main body, individuals with the top 20% genetic distance from population B were added;
(4) A+10%C: With population A as the main body, individuals with the top 10% genetic distance from population C were added;
(5) A+15%C: With population A as the main body, individuals with the top 15% genetic distance from population C were added;
(6) A+20%C: With population A as the main body, individuals with the top 20% genetic distance from population C were added.

The genetic distance screening strategy, based on multidimensional scaling (MDS), was used to calculate the genetic distances between PopA and PopB/C, respectively. Individuals in PopB/C were screened according to the closest genetic distances, retaining the top 10%, 15%, and 20% of individuals with genetic similarity to PopA. Reference populations were constructed separately to evaluate the accuracy of genomic prediction across/within breeds under different screening thresholds and to identify the optimal proportion of introduction. These calculations were completed using PLINKv1.9 software.

### 2.4. Model and Analysis

In this study, the statistical methods used to estimate breeding values include the genomic best linear unbiased prediction (GBLUP) model, the single-step genomic best linear unbiased prediction (ssGBLUP) model, and the weighted genomic best linear unbiased prediction (wGBLUP) model based on genomic information.

**Genomic best linear unbiased prediction**

In parameter estimation using genomic information for GBLUP, a general linear mixed model was employed, and the model was constructed using the HIBLUP_1.4.0 software. Although GBLUP and PBLUP used the same fixed effects, GBLUP employed a genomic relationship matrix (GRM) based on SNP markers to estimate genomic estimated breeding values (GEBVs). The expression for GRM is as follows:

(3) $$G=\frac{MM^{\prime }}{\sum_{i=1}^{m}{2p}_{i}(1-p_{i})}$$

Here, *M* is the matrix of individual genes (where homozygotes, heterozygotes, and alternative homozygotes are converted to 0, 1, and 2, respectively), m is the total number of SNP markers, and pi is the frequency at the i-th position in the SNP.

Then, since only additive genetic effects are modeled, only these effects are considered here:

(4) $$Var\left(a\right)=G\sigma_{g}^{2}$$

The general linear mixed model equation for GBLUP is as follows:

(5) y=Xb+Za+e

(6) $$\left[\begin{array}{cc} X^{\prime }X & X^{\prime }Z\\ Z^{\prime }X & Z^{\prime }Z+\lambda G^{-1} \end{array}\right]\left[\begin{array}{c} \hat{b}\\ \hat{g} \end{array}\right]=\left[\begin{array}{c} X^{\prime }y\\ Z^{\prime }y \end{array}\right]$$

where *y* is the vector of phenotypes, *X* is the matrix associating fixed effects with each animal individual, *b* is the vector of fixed effects, *Z* is the design matrix allocating records to genetic values, *g* is the vector of additive genetic effects for individuals, *G* is the genomic relationship matrix, *e* is the vector of residual error effects with a normal distribution ~N(0, $G\sigma_{e}^{2}$), and $\sigma_{e}^{2}$ is the residual variance. $\lambda =\sigma_{e}^{2}/\sigma_{g}^{2}$.

**Single-step Genomic Best Linear Unbiased Prediction**

In the ssGBLUP method, the statistical model is similar to that used in traditional evaluation. However, both non-genotyped and genotyped animals are simultaneously included in the hybrid relationship matrix H, which is a combination of the A (numerator relationship matrix) and G (genomic relationship matrix) matrices. In this case, the $A^{-1}$ matrix is replaced by the $H^{-1}$ matrix derived by Aguilar et al. [27], the expression of which is as follows:

(7) $$H^{-1}=A^{-1}+\left[\begin{array}{cc} 0 & 0\\ 0 & G^{-1}-A_{22}^{-1} \end{array}\right]$$

where $H^{-1}$ is the inverse of the realized relationship matrix that combines the pedigree and genomic information, $G^{-1}$ is the inverse of the genomic relationship matrix, and $A_{22}^{-1}$ is the inverse of the pedigree-based relationship matrix for genotyped animals. The G matrix is obtained according to model (5).

**Weighted Best Linear Unbiased Prediction**

In the weighted GBLUP, the model and inference are the same, except for the use of a different SNP effect vector when constructing the G matrix. By the SLEMM software (v0.90.1) [28], two schemes for optimizing genomic prediction by SNP weighting are provided, namely, based on (1) the minor allele frequency (MAF) dependence of SNP effect sizes and (2) the SNP effect estimates with weight W equal to the identity matrix.

SLEMM fits the following linear mixed model:

(8) $$y=X\beta +Z\alpha +e\;\alpha \sim N(0,W\sigma_{\alpha }^{2}),\;e\sim N(0,R\sigma_{e}^{2})$$

where *y* is a vector of phenotypes for a quantitative trait, *β* is a vector of fixed effects, including the mean, *X* is the design matrix for *β*, *α* is a vector of SNP effects with a diagonal covariance matrix $W\sigma_{\alpha }^{2}$, *Z* is a matrix of standardized genotypes, and *e* is a vector of residuals with a diagonal covariance matrix $R\sigma_{e}^{2}$. R is usually equal to an identity matrix, and the diagonal elements of *W* are weights, with a mean representing the relative contribution of the SNP effect to the genetic variance; that is, *Wj* represents the contribution of SNP j to the genetic variance.

Due to linkage disequilibrium (LD), the effect of a quantitative trait locus (QTL) can be captured by nearby SNP loci, and the effects of adjacent SNPs tend to be similar in model fitting. Therefore, the second SNP weighting scheme is used in this study, with the formula as follows:

(9) $$W_{jj}=C\cdot \frac{1}{2S+1}\sum_{k=j-S}^{j+S}\hat{\alpha_{k}^{2}}$$

where *C* is a scaling constant to maintain a mean weight of 1, *S* is the number of SNPs on each side of SNP j, and $\hat{\alpha_{k}}$ is the estimate of the effect of the kth SNP in an existing BLUP with *W* equal to the identity matrix. This specification of the jth SNP’s weight borrows information from a window of 2S+1 SNPs. SLEMM first fits the model with training data where *W* is equal to the identity matrix and is then fitted with *W*, computed by Equation (9).

### 2.5. Accuracy of Genomic Prediction

In this study, model performance was evaluated using prediction accuracy and unbiasedness. Since the true breeding values (TBVs) were directly provided in the simulation, prediction accuracy is defined as the Pearson correlation coefficient between the genomic estimated breeding values (GEBVs) and TBVs, calculated as:

Accuracy = Corr(GEBV,TBV),(10)

where Corr ranges from 0 to 1, indicating the strength of the linear relationship between GEBVs and TBVs.

## 3. Results

### 3.1. Population Genetic Characterization

**Figure 2:**
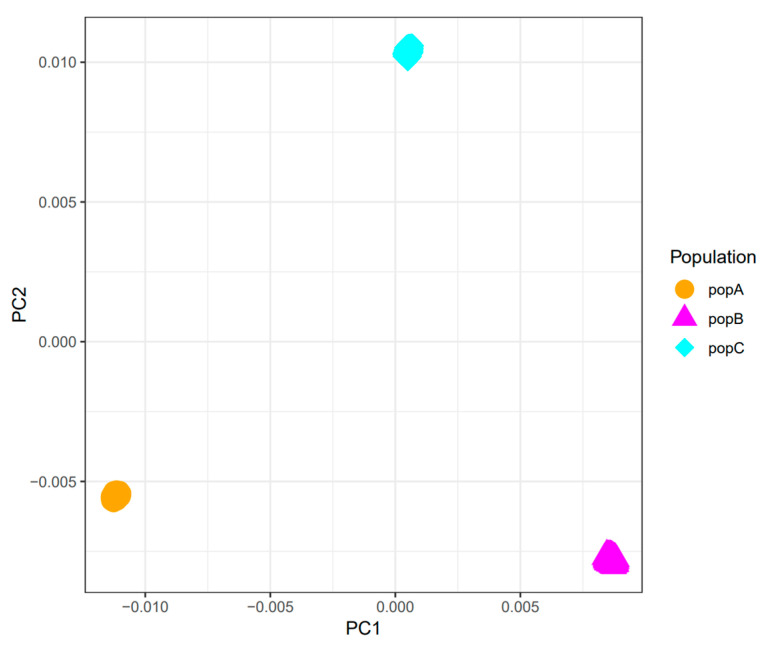
Principal Component Analysis (PCA) of three distinct populations (PopA, PopB, and PopC). The *x*-axis represents Principal Component 1 (PC1), and the *y*-axis represents Principal Component 2 (PC2). Different colors and shapes of points indicate different populations, with orange circles representing PopA, dark pink triangles representing PopB, and cyan diamonds representing PopC.

We used PCA to characterize the genetic structure of three populations (PopA, PopB, and PopC). PC1 and PC2 together explain most of the variation (Figure 2). The three populations are clearly separated along these axes, indicating substantial genetic differences. The divergence between PopA and PopB may reflect distinct key genetic features, whereas PopC’s isolated position suggests a unique ancestry.

**Figure 3:**
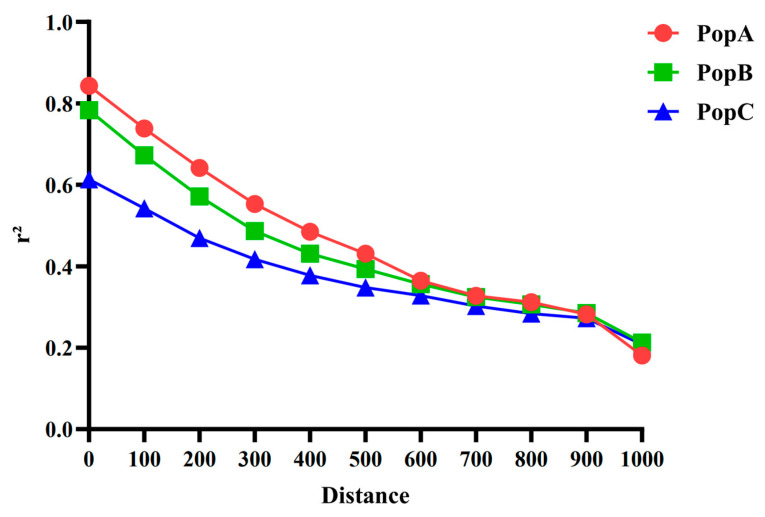
Linkage disequilibrium (LD) among markers in the most recent generation (Generation 10) of three distinct populations (PopA, PopB, and PopC). The *x*-axis shows the range of distances between markers in the genome, measured in kilobases (kb); the *y*-axis shows the average LD, represented by r^2^.

We examined LD decay (measured as r^2^) in all three groups (Figure 3). In every population, r^2^ declines with increasing distance, but the rate differs: decay is slowest in PopA (highest r^2^) and fastest in PopC (lowest r^2^). Differences are pronounced at short ranges (0–500 kb) and converge as the distance approaches 1 Mb [29].

**Figure 4:**
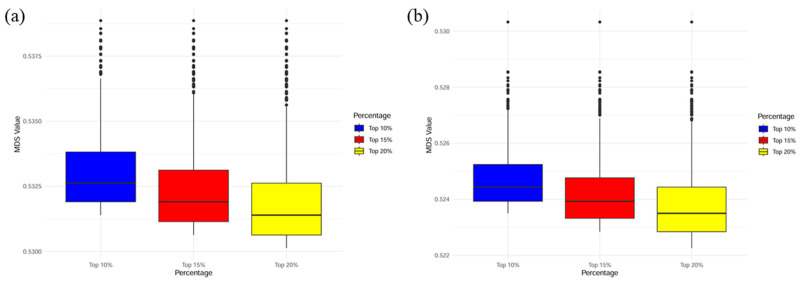
Multidimensional scaling (MDS) values between individuals of PopA and PopB/C at the last generation (Generation 10) for different levels of genetic distance. (**a**) MDS values between PopA and PopB individuals at different levels of genetic distance. (**b**) MDS values between PopA and PopC individuals at different levels of genetic distance. The *x*-axis shows the percentage of different levels of genetic distance; the *y*-axis shows the MDS values.

Multidimensional scaling (MDS) was applied to quantify genetic divergence between PopA and PopB/C at three distance thresholds (top 10%, 15%, and 20%) (Figure 4). MDS values rise with the percentile, indicating greater differentiation. For example, PopA vs. PopB diverges most at the top 20% level. Moreover, at the same threshold, the magnitude of PopA vs. PopC can differ from PopA vs. PopB (e.g., slightly larger at the top 10% but smaller at the top 20%). These patterns demonstrate that genetic architecture affects predictive accuracy and that this influence intensifies as genetic distance increases.

### 3.2. Impact of Different Genetic Distance Levels on the Accuracy of GEBV Prediction

**Figure 5:**
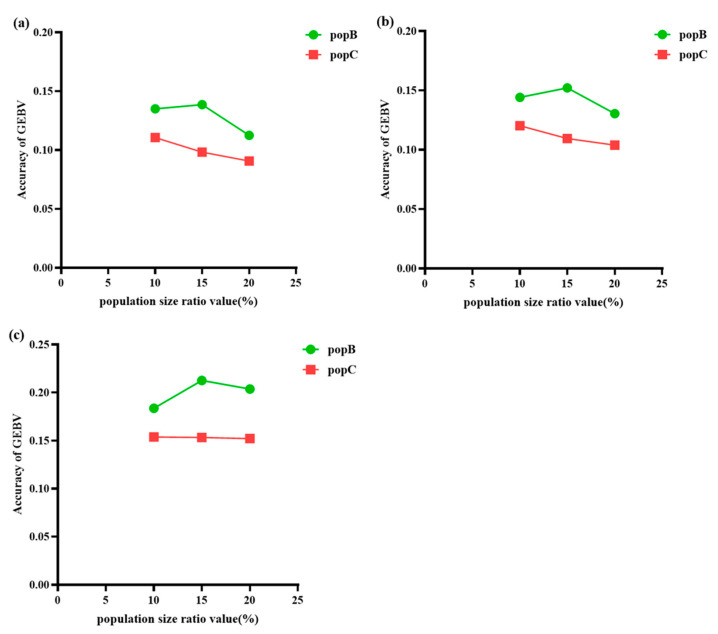
Comparison of accuracy based on the GBLUP (**a**), ssGBLUP (**b**), and wGBLUP (**c**) evaluation models between PopA and PopB/C at different levels of genetic distance. The *x*-axis shows the percentage of different genetic distance population sizes; the *y*-axis shows the accuracy of GEBVs.

This study aims to compare the accuracy of genomic selection prediction in small populations through multi/cross-breed evaluation under different levels of genetic distance between PopA and PopB/C. Specifically, the overall results based on the GBLUP evaluation model in population A combined with different proportions of genetically close PopB/C (Figure 5) indicate that the accuracy of predicting PopB/C varies at different genetic distance percentage levels, and there are also differences in accuracy between different populations at the same level. For PopB, which has a relatively stable LD decay rate, the results show that the accuracy of GEBVs first increases and then decreases with the increasing proportion of PopB in the reference population, peaking at 15% (0.139). This suggests that mixing a moderate proportion of PopB individuals in the reference population may optimize the prediction accuracy of GEBVs, but too many PopB individuals may introduce noise and reduce prediction accuracy. In contrast, for PopC, which has a faster LD decay rate, the accuracy shows a continuous downward trend with the increasing mixing proportion. This indicates that mixing a higher proportion of PopC individuals in the reference population may not be conducive to the accuracy of GEBVs. The reason may be that the rapid LD decay of PopC reduces the information available for genomic prediction, thereby limiting its contribution to improving prediction accuracy. Even when the proportion of PopC is low, this rapid LD decay limits the improvement of GEBV accuracy by PopC, as shown in Figure 5, where the GEBV accuracy of PopC is always lower than that of PopB.

Moreover, there are also significant differences in accuracy between PopB and PopC at the same percentage level. For example, at the 10% level, the accuracy of PopB (0.135) is higher than that of PopC (0.111), and this trend continues at the 15% and 20% levels, with PopB’s accuracy (0.139 and 0.113) always being better than PopC’s accuracy (0.099 and 0.091). These findings suggest that the genetic structure and relatedness between PopA and PopB/PopC play a key role in determining the accuracy of GEBVs, and the optimal percentage level of including individuals from different populations in the reference population may vary depending on the specific population.

When analyzing population A combined with different proportions of PopB and PopC using the ssGBLUP evaluation model, the overall trends shown in Figure 5 are similar to those obtained with the GBLUP model. There are differences in the accuracy of predicting PopB and PopC at different genetic distance percentage levels, and accuracies also vary between populations at the same level. Specifically, for PopB, which has a slower LD decay, the accuracy of GEBVs first increases and then decreases with the increasing proportion of PopB in the reference population, peaking at 15% with an accuracy of 0.152. This suggests that an appropriate proportion of PopB in the reference population may help enhance the prediction accuracy of GEBVs, while a higher proportion may introduce noise and reduce accuracy. In contrast, for PopC, which has a faster LD decay, the accuracy continuously decreases with the increasing proportion, indicating that a higher proportion of PopC in the reference population may not be beneficial for the accuracy of GEBVs. This is likely because the rapid LD decay of PopC reduces the amount of information available for genomic prediction, limiting its positive contribution to prediction accuracy. Even at a lower proportion, the rapid LD decay of PopC restricts its ability to improve GEBV accuracy, as shown in Figure 5b, where the GEBV accuracy of PopC is always lower than that of PopB.

Moreover, there are significant differences in accuracy between PopB and PopC at the same proportion level. For example, at the 10% proportion, the accuracy of PopB is 0.144, higher than PopC’s 0.120, and this trend continues at the 15% and 20% proportions, with PopB’s accuracies (0.152 and 0.131, respectively) always being higher than those of PopC (0.114 and 0.102, respectively). These results indicate that the genetic structure and relatedness between population A and PopB/PopC have important impacts on the accuracy of GEBVs, and the optimal proportions of individuals from different populations in the reference population may vary depending on the specific population.

When analyzing population A combined with different proportions of PopB and PopC at various genetic distances using the wGBLUP evaluation model, the trends observed in Figure 5 are essentially consistent with the results from the GBLUP and ssGBLUP models. It can be seen that the accuracy of predicting PopB and PopC varies at different genetic distance percentage levels, and there are also differences in accuracy between populations at the same genetic distance level. Specifically, for PopB, which has a slower genetic distance decay, the accuracy of GEBVs first increases and then decreases with the increasing proportion of PopB in the reference population, peaking at 15% with an accuracy of 0.214. This suggests that including an appropriate proportion of PopB individuals in the reference population may help improve the prediction accuracy of GEBVs, but a higher proportion may introduce noise and reduce prediction accuracy. In contrast, for PopC, which has a faster genetic distance decay, the accuracy continuously decreases with the increasing proportion in the reference population, indicating that including a higher proportion of PopC individuals may not be beneficial for the accuracy of GEBVs. This is likely because the rapid genetic distance decay of PopC reduces the amount of information available for genomic prediction, thereby limiting its contribution to improving prediction accuracy. Even at a lower proportion, the rapid genetic distance decay of PopC restricts its ability to enhance GEBV accuracy, as shown in Figure 5c, where the GEBV accuracy of PopC is always lower than that of PopB.

Moreover, there are significant differences in accuracy between PopB and PopC at the same genetic distance percentage level. For example, at the 10% genetic distance level, the accuracy of PopB is 0.184, higher than PopC’s 0.154, and this trend continues at the 15% and 20% genetic distance levels, with PopB’s accuracies (0.214 and 0.204, respectively) always being higher than those of PopC (0.153 and 0.152, respectively). These results indicate that the genetic structure and relatedness between population A and PopB/PopC play a key role in determining the accuracy of GEBVs, and the optimal proportions of individuals from different populations in the reference population may vary depending on the specific population.

### 3.3. Comparison of Prediction Accuracy of Three Evaluation Models at Different Genetic Distance Levels

**Figure 6:**
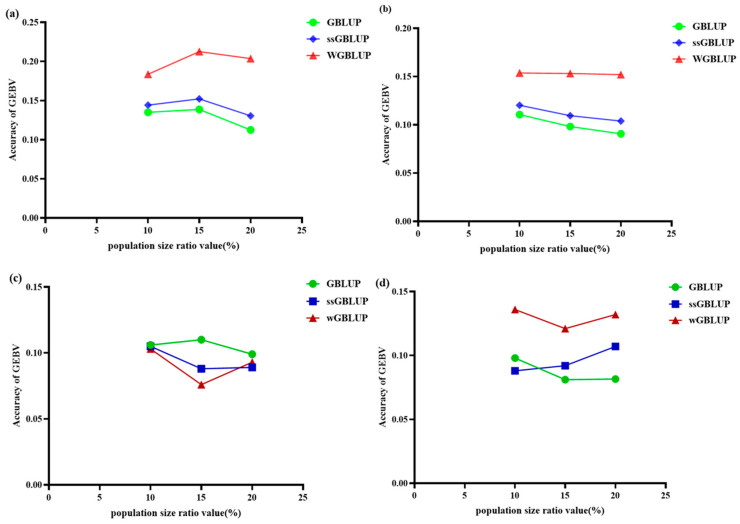
Comparison of the accuracy of GEBV prediction for PopA combined with PopB/C at different genetic distance levels using three evaluation models (GBLUP, ssGBLUP, and wGBLUP). (**a**) Comparison of the accuracy of different models at various genetic distance levels between PopA and PopB. (**b**) Comparison of the accuracy of different models at various genetic distance levels between PopA and PopC. (**c**) Comparison of model accuracies for PopB at different levels of genetic distance. (**d**) Comparison of model accuracies for PopC at different levels of genetic distance. The *x*-axis shows the percentage of population size at different genetic distance levels; the *y*-axis shows the accuracy of GEBVs.

When comparing the genomic selection prediction accuracy of reference populations composed of PopA combined with different mixing proportions of PopB/C at various genetic distance levels for multi/cross-breed evaluation of populations with different LD (linkage disequilibrium) decay patterns, it was observed that the ssGBLUP (single-step genomic best linear unbiased prediction) and wGBLUP (weighted genomic best linear unbiased prediction) methods exhibited higher accuracy in predicting PopB with moderate LD decay patterns, although there were significant differences between the two methods (Figure 6a and Table S1). For instance, when PopA was combined with PopB at a genetic distance level of 10%, the accuracy of the GBLUP (genomic best linear unbiased prediction) model was 0.135, that of the ssGBLUP model was 0.144, and that of the wGBLUP model was 0.184, with the wGBLUP model showing the highest accuracy. Similarly, at a genetic distance level of 15%, the accuracy of the GBLUP model was 0.139, the ssGBLUP model’s accuracy was 0.152, and the wGBLUP model’s accuracy was 0.213. At a genetic distance level of 20%, the accuracy of the GBLUP model was 0.113, the ssGBLUP model’s accuracy was 0.131, and the wGBLUP model’s accuracy was 0.204. These findings indicate that when combining populations at genetic distance levels of 10%, 15%, and 20%, the wGBLUP model outperformed the GBLUP and ssGBLUP models in terms of accuracy, especially at the genetic distance level of 15%, where the highest accuracy was achieved.

Further analysis revealed that when population A was combined with population C at different genetic distance levels, the results were similar to those observed with population B (Table 2). Specifically, at a genetic distance level of 10%, the accuracy of the GBLUP, ssGBLUP, and wGBLUP models was 0.111, 0.120, and 0.154, respectively, with the wGBLUP model performing the best. At a genetic distance level of 15%, the accuracy of the GBLUP, ssGBLUP, and wGBLUP models was 0.099, 0.109, and 0.153, respectively, with the wGBLUP model still leading. At a genetic distance level of 20%, the accuracy of the GBLUP, ssGBLUP, and wGBLUP models was 0.091, 0.104, and 0.152, respectively, with the wGBLUP model maintaining the highest accuracy. These results demonstrate that at genetic distance levels of 10%, 15%, and 20%, the wGBLUP model consistently outperformed the GBLUP and ssGBLUP models, with the highest accuracy achieved at the genetic distance level of 15% (Figure 6b).

Furthermore, when comparing the genomic prediction accuracies of single-breed versus multi-breed reference populations across different genetic distance levels for PopB and PopC, we observed that blending breeds consistently yielded higher accuracies than single-breed evaluations (Figure 6). For PopA + PopB at the 10% genetic distance level, accuracies were 0.135 (GBLUP), 0.144 (ssGBLUP), and 0.184 (WGBLUP), whereas the accuracies for PopB alone were 0.106, 0.105, and 0.103, respectively—demonstrating the superiority of the mixed reference. At the 15% level, the mixed reference achieved 0.139 (GBLUP), 0.152 (ssGBLUP), and 0.213 (WGBLUP) accuracies, while PopB alone produced accuracies of 0.110, 0.088, and 0.076. Similarly, at 20%, the mixed reference yielded accuracies of 0.113 (GBLUP), 0.131 (ssGBLUP), and 0.204 (WGBLUP), compared to 0.099, 0.089, and 0.093 in the single-breed PopB reference. These results indicate that, across all tested genetic distance levels (10%, 15%, and 20%), multi-breed reference populations outperformed their single-breed counterparts in predictive ability. As shown in Figure 6, PopB and PopC exhibit the same trend: for all three genetic distance levels (10%, 15%, and 20%), multi-breed reference panels consistently deliver higher prediction accuracies than single-breed reference panels.

In summary, our results indicate that, under limited reference population sizes, the WGBLUP model tends to yield higher prediction accuracies for populations with differing LD decay patterns, particularly at a 15% genetic distance threshold. Whether combined with PopB or PopC, WGBLUP outperforms both GBLUP and ssGBLUP, underscoring the importance of accounting for each population’s genetic structure and LD pattern when assembling the reference set.

## 4. Discussion

The accuracy of genomic estimated breeding values (GEBVs) is influenced by several key factors, including the heritability level of the target trait [30], the size of the reference population [5,31,32], the density of genotypic markers [33], the effect sizes and number of quantitative trait loci (QTL) [21], and the degree of linkage disequilibrium (LD) between markers and QTL [34]. In multi-breed genomic prediction in beef cattle, improvements in accuracy are constrained by several factors, with the construction of the reference population being particularly critical—it not only directly affects the application of genomic selection (GS) technology but also has a positive correlation with prediction accuracy in terms of its size and genetic diversity [35]. To overcome the limitations of sample size on prediction accuracy, several optimization strategies have been proposed in current research, for example, integrating multiple populations from different breeding organizations to expand the effective reference population or leveraging genetic similarities between breeds to construct composite reference populations to enhance genetic variation coverage. This multi-population joint analysis approach can effectively increase the genetic diversity of the reference population, thereby improving the stability and reliability of genomic prediction. Studies using the ssGBLUP (single-step genomic best linear unbiased prediction) method to combine purebred and crossbred data in reference populations have significantly improved prediction accuracy. This method not only fully utilizes genetic information from different breeds but also enhances the robustness and reliability of evaluation by integrating large-scale datasets [4]. Van den Berg et al. [36] reported that integrating a composite reference population that includes crossbred individuals and balances the contributions of different breeds is crucial for significantly improving the accuracy of genomic prediction for both purebred and crossbred animals. Key elements include balancing genetic diversity, optimizing the LD structure, and prioritizing the use of causal variants shared across different breeds. In this study, the crucial role of composite breed reference populations in improving the accuracy of multi-breed beef cattle genomic prediction was further confirmed, especially when the population includes crossbred offspring and balanced breed contributions. Compared to a single dominant breed reference population, crossbred/other breed individuals can optimize the LD structure and enhance the ability to capture QTL shared across breeds [11,37]. However, it is important to note that over-reliance on a single breed can reduce prediction accuracy for minor breeds, indicating that balancing genetic contribution proportions (with a suggested crossbred individual proportion of 20–40%) is a core principle in designing composite reference populations. Therefore, this study explored the accuracy of multi/cross-breed genomic prediction in multi-breed beef cattle populations at different genetic distance levels using three evaluation methods (GBLUP, ssGBLUP, and wGBLUP) to compare the accuracy of constructing composite reference populations with different screening thresholds and to identify the optimal mixing proportions.

In multi-breed genomic prediction, constructing composite reference populations can effectively enhance prediction accuracy, particularly for breeds with smaller reference population sizes. However, the effectiveness is significantly influenced by the genetic distance between breeds and the proportion of each breed in the population. Multiple studies have shown that integrating data from multiple breeds can enhance the prediction ability for breeds with smaller populations, but it is necessary to balance the proportion of each breed to avoid interference from larger populations on the prediction accuracy of smaller ones. Among these factors, genetic distance is a key limiting factor for across-breed prediction—predictions tend to be more accurate between closely related breeds, while predictions often fail between breeds that have diverged for more than 200 years [12,38]. Additionally, when the genetic correlation between breeds is low, models that account for breed origin can more effectively utilize across-breed genetic information. The advantage of across-breed evaluation lies in integrating genetic resources from multiple breeds to improve the robustness of molecular breeding values (MBVs). Although its predictive ability for untrained breeds is limited, it can serve as a transitional solution for establishing breed-specific prediction models. These findings provide important evidence for optimizing multi-breed genomic selection strategies, emphasizing the need to comprehensively consider factors such as genetic distance, population proportion, and model selection [14]. The results of this study indicate that combining populations can improve the accuracy of multi/cross-breed evaluation. However, when data from different breeds are integrated into a single reference population, prediction accuracy tends to decrease with increasing genetic distance levels. Nevertheless, we found that optimizing the proportion of breeds can mitigate this trend to some extent. Specifically, when the proportion of genetic distance between PopA and PopB in the composite reference population reached 15%, the prediction accuracy was the highest. However, when the genetic distance proportion reached 20%, the accuracy decreased. This finding suggests that, despite the limitations of across-breed evaluation in predicting untrained breeds, adjusting the proportion of individuals in the reference population that are genetically close to the main breed can significantly enhance its predictive ability. Furthermore, Wientjes et al. [6] conducted a study on the impact of QTL characteristics on the accuracy of multi-breed genomic prediction. The results showed that individuals with closer genetic relationships share more genomic segments, making the LD (linkage disequilibrium) relationship between markers and QTL more similar among these individuals, which, in turn, facilitates more accurate prediction of breeding values for candidate individuals. For example, when the reference population includes individuals from the same breed as the candidate individuals, the prediction accuracy is usually higher than that of a reference population containing only individuals from other breeds. This confirms the conclusions drawn in this study.

Additionally, in this study, we found that the wGBLUP model exhibited higher accuracy in multi/cross-breed evaluation compared to the ssGBLUP and GBLUP models. This finding has been partially reflected in the literature on single-breed evaluations, where some studies have indicated that the wGBLUP model can achieve higher accuracy than the ssGBLUP and GBLUP models in single-breed contexts. For instance, Zhao et al. [39] demonstrated that in multi-breed evaluations, the wGBLUP model significantly improved prediction accuracy by accounting for the heterogeneity of allele frequencies between different populations, particularly in populations with smaller sample sizes, where it outperformed the traditional GBLUP method. Moreover, ssGBLUP tends to show more pronounced advantages in populations with smaller sample sizes. However, as the number of genotyped individuals in the population increases, the advantage of ssGBLUP diminishes and becomes less than that of wGBLUP. This may be because the wGBLUP model considers the impact of SNP allele frequencies on weights and employs a window weighting scheme to adjust the weights of current SNPs based on the effect estimates of neighboring SNPs. This approach effectively identifies SNPs with similar linkage disequilibrium phases across multiple breeds and estimates their effects individually, thereby fully utilizing the diversity of genetic information and capturing genetic variation within breeds more precisely [40]. The superior performance of the wGBLUP model in this study is likely due to its optimization of prediction by assigning weights to each SNP in the genomic relationship matrix, which may enhance prediction accuracy. However, differences in genetic background, population structure, population size and quality, and potential gene flow between different breeds can all influence the model’s estimation of genetic effects. Whether its advantage can be stably maintained requires further validation through additional studies.

It is important to acknowledge that the findings of this study have certain limitations, which may affect the generalizability and applicability of the conclusions. In terms of the design of mixed reference populations, the current exploration only covers specific genetic distance intervals and three mixing ratios (10%, 15%, and 20%), without including a broader range of proportional gradients (such as a continuous gradient from 5% to 30%) or addressing dynamic evolutionary processes like long-term gene flow and population bottlenecks. This could restrict the direct application of the conclusions to natural populations or complex breeding systems (e.g., multi-generational cross-breeding populations). For instance, when there is frequent gene flow among populations, the pattern of how mixing ratios affect prediction accuracy may differ from the results observed in this study, and this hypothesis requires further validation in subsequent research. In terms of analyzing the mechanisms underlying the model’s advantages, the current study primarily focuses on LD decay patterns and marker weighting effects. However, in practical breeding, complex mechanisms such as gene-environment interactions (e.g., differences in feeding and management practices) and spatiotemporal-specific regulation of gene expression may influence genomic prediction accuracy by affecting phenotypic variation of traits. These dimensions have not been thoroughly analyzed in this study, meaning that the current explanation for the advantages of models like wGBLUP remains limited to linkage effects at the genomic level and has not yet been fully connected to the mechanisms of phenotypic formation in practical breeding. Furthermore, the conclusions of this study are based on the genetic background of specific beef cattle breeds, with their LD decay characteristics and population structure exhibiting species specificity. For species with greater genetic distance (e.g., sheep and pigs) or other beef cattle breeds with significantly different genetic backgrounds, the optimal threshold for mixing ratios and the priority of model application may change. Thus, the generalizability of the conclusions requires validation across species and breeds. Despite these limitations, this study still provides critical insights for genomic selection in multi-breed mixed reference populations. Future research can advance in three directions: expanding the gradient range of mixing ratios and simulating long-term breeding scenarios using population dynamic evolution models; integrating multi-omics data (such as transcriptomics and metabolomics) to analyze the impact of gene–environment interactions on prediction accuracy; and conducting cross-species validation studies to clarify the applicable boundaries of the conclusions. These efforts will help improve the theoretical system of multi-breed genomic selection and provide more precise guidance for practical breeding.

## 5. Conclusions

In summary, our study highlights that when incorporating additional populations into genetic evaluation models to improve predictive accuracy, the mixing proportion between populations matters—particularly for local beef cattle breeds with divergent genetic structures and LD patterns and limited resources. Under restricted reference population sizes, a 15% mixing proportion led to a significant (*p* = 0.042) increase in wGBLUP accuracy compared to a 10% proportion. Relative to GBLUP and ssGBLUP, wGBLUP tended to perform better when combined with PopB or PopC at this 15% level. At 10% or 20% mixing, none of the three models outperformed the 15% scenario, although wGBLUP and ssGBLUP remained comparatively stable. These findings underscore the need to consider mixing proportion, genetic distance, and model choice in cross-breed evaluations, especially when resources are limited. This study still has noteworthy limitations: while genetic distance and LD patterns were analyzed, more detailed population stratification analyses (e.g., admixture or Fst) could help better elucidate the mechanisms underlying the effects of mixing proportions. Additionally, genotype-by-environment interactions (G×E) were not considered, which may affect prediction accuracy in practical breeding applications. Therefore, future research should focus on developing dynamic optimization strategies for mixing proportions, integrating multi-omics data to improve model robustness, and validating the method’s applicability in larger and more diverse populations. These improvements will help advance the practical application of cross-population genomic prediction in breeding practices.

## Figures and Tables

**Table 1:** Parameters of the simulation process.

Population Structure	PopA	PopB	PopC
Step 1: Historical Generations (HG)			
Number of generations in phase 1 (size)	0 (200)	0 (500)	0 (1000)
Number of generations in phase 2 (size)	1000 (200)	1000 (200)	1000 (1000)
Number of generations in phase 3 (size)	1095 (1000)	1095 (1000)	1095 (200)
Step 2: Recent Generations (RG)			
Number of founder males from HG	200	220	200
Number of founder females from HG	2800	2335	2800
Selection and mating	ebv/h		
Sire/Dam replacement	0.6/0.3	0.5/0.3	0.5/0.2
Mating system	Random		
Culling design	ebv/L		
Genome			
Number of chromosomes	29 (no X Chr)		
Genome length	2715.85 cm		
Number of markers	58,990 (50 k)		
Marker/QTL positions	Random		
Number of marker/QTL alleles	2/2 3 4		
Marker of allele frequencies	Equal		
QTL allele effects	Equal		
Mutation rate	2.5 × 10^5^		

**Table 2:** Comparison of the accuracy of GEBV prediction for PopA combined with PopB/C at different genetic distance levels using three evaluation models (GBLUP, ssGBLUP, and wGBLUP).

	Population	GBLUP		ssGBLUP		wGBLUP	
Percentage		PopB	PopC	PopB	PopC	PopB	PopC
10%		0.135	0.111	0.144	0.120	0.184	0.154
15%		0.139	0.099	0.152	0.109	0.213	0.153
20%		0.113	0.091	0.131	0.104	0.204	0.152

## Data Availability

The original contributions presented in this study are included in the article; further inquiries can be directed to the corresponding author.

## Supplementary Materials

The following supporting information can be downloaded at: https://www.mdpi.com/article/10.3390/ani15162463/s1, Table S1: Summary of standard deviations, mean differences, and significance results based on GBLUP, ssGBLUP, and wGBLUP methods for populations B and C.

## Abbreviations

The following abbreviations are used in this manuscript:

GS	Genomic selection
TBV	True breeding value
EBV	Estimation of breeding value
BLUP	Best linear unbiased prediction
GEBV	Genomic estimated breeding value
SNP	Single-nucleotide polymorphism
MAF	Minor allele frequency
LD	Linkage disequilibrium
QTL	Quantitative trait loci
GBLUP ssGBLUP	Genomic best linear unbiased prediction Single-Step genomic best linear unbiased prediction
wGBLUP	Weighted best linear unbiased prediction
GRM	Genomic relationship matrix
